# Fecal Microbiota Transplantation in Recurrent Clostridium Difficile Infection: Is it Superior to Other Conventional Methods?

**DOI:** 10.7759/cureus.9653

**Published:** 2020-08-11

**Authors:** Zayar Lin, Zafar Iqbal, Juan Fernando Ortiz, Sawleha Arshi Khan, Nusrat Jahan

**Affiliations:** 1 Internal Medicine, California Institute of Behavioral Neurosciences and Psychology, Fairfield, USA; 2 Emergency Medicine, California Institute of Behavioral Neurosciences and Psychology, Fairfield, USA; 3 Emergency Department, The Kidney Center, Karachi, PAK; 4 Neurology, California Institute of Behavioral Neurosciences and Psychology, Fairfield, USA; 5 Research, California Institute of Behavioral Neurosciences and Psychology, Fairfield, USA

**Keywords:** clostridium difficile infection, clostridium difficile infection treatment, fecal microbiota transplantation in clostridium difficile infection

## Abstract

*Clostridium difficile *(*C. difficile*) is a gram-positive species of spore-forming bacteria. *C. difficile *infection (CDI) is one of the most common hospital-acquired infections in the United States, mainly caused by the use of recent antibiotics that leads to intestinal dysbiosis. Recurrent *C. difficile* infection (rCDI) often occurs after the successful treatment of CDI. Approximately, 30% of patients experience a clinical recurrence of prior symptoms within eight weeks of antibiotic cessation. This present literature review covers the current pathophysiology of CDI, risk factors for infection, diagnostic methods, several treatment modalities, and the potential use of fecal microbial transplant (FMT) for patients with multiple recurrent CDIs. Recent studies have focused on FMT, with an efficacy rate of nearly 90% in multiple recurrent CDI settings. Despite its efficacy, it is not commonly used as first-line treatment. More studies are needed to establish this therapy as the first option in patients with rCDI.

## Introduction and background

*Clostridium difficile* (*C. difficile*) is a gram-positive, anaerobic, spore-forming bacillus that was retitled to *Clostridioides difficile* in 2016 [1]. *C. difficile* infection (CDI) is one of the most common nosocomial diseases in the United States, with approximately 453,000 infections and 29,000 deaths in 2011 [2]. It has been a big problem for U.S healthcare systems, leading to roughly $5 billion in healthcare costs [3].

The incidence and fatality rate of recurrent *C. difficile* infection (rCDI) have been escalating worldwide due to an increase in drug-resistant strains [4]. rCDI is generally described as an episode of CDI that occurs within eight weeks of the previous event [5,6], which can be due to relapse of the same strain or a different strain [7]. In some reported studies, rCDI occurred in approximately 15-30% of patients who initially responded to antibiotic therapy [8,9].

The pathogenesis of CDI supports the hypothesis that antibiotic use can change the constitution and homeostasis of intestinal microbiota, which leads to pathogenic toxin-producing *C. difficile* strains to inhabit the intestine, causing illness ranging in severity from mild diarrhea to pseudomembranous colitis, toxic megacolon, and sepsis [10,11].

Over the last few years, fecal microbiota transplantation (FMT), which consists of fecal microbiota infusion from a healthy donor into a recipient subject, has been more successful and durable than conventional therapy for rCDI patients. Particularly fresh donor FMT can reconstitute the standard composition of intestinal microbiota in patients and restore their function to protect against further CDI recurrence and colonization [12-15]. FMT was conducted in hundreds of patients, and the findings have been published in the medical literature. Besides, FMT is successfully treated in 87% of rCDI patients [16].

Our article aims to include high-quality papers on this topic for more detailed research to be updated to contribute to some significant assistance for clinical practice.

## Review

Method

Data is collected from PubMed using regular keywords and MeSH strategy and subheadings. Table 1 demonstrates regular and MeSH keywords for the literature search.

**Table 1:** Regular and MeSH keywords for literature search

Regular Keyword	Clostridium difficile infection
Total records	34805
Records selected	1753

Regular Keyword	Clostridium difficile infection treatment
Total records	20622
Records selected	2149

MeSH Keyword	Fecal microbiota transplantation in Clostridium difficile infection
Total records	668
Records selected	263

Studies were selected after applying the following inclusion/exclusion criteria: The inclusion criteria were: (1) studies involving only human subjects; (2) papers published in the English language and within the past 10 years; (3) all types of studies including observational studies, clinical trials, randomized controlled trials, cohort study, and review articles; and (4) papers available as free full-text format. The exclusion criteria were (1) animal studies; (2) papers published in languages other than English; (3) meta-analyses, case reports, and case series studies.

Results

Table 2 indicates the total number of articles after inclusion/exclusion criteria have been used in the following order:

**Table 2:** Total number of articles after applying inclusion/exclusion criteria

Regular Keyword	Clostridium difficile infection
Total records	34805
Inclusion/exclusion criteria	
Humans	27412
English language	20432
Published within 10 years	7743
Full text online	1753

Regular Keyword	Clostridium difficile infection treatment
Total records	20622
Inclusion/exclusion	
Humans	17260
English language	13107
Published within 10 years	5335
Full text online	2149

MeSH Keyword	Fecal microbiota transplantation in Clostridium difficile infection
Total records	668
Inclusion/exclusion	
Humans	658
English language	599
Published within 10 years	598
Full text online	263

A total of 1490 articles from keyword search "CDI were removed due to lack of outcome of interest in “CDI treatment” and the elimination of duplicates. After a detailed search, the total number of articles collected was 263 free full texts. All 263 free full texts were reviewed, and 233 were removed due to one of the following reasons: (1) not specifying the disease of interest (those which did not assess CDI separately but were instead a composite assessment of CDI with other *Clostridium* species); (2) case report or case series studies (as it only assessed for a particular patient in focus; (3) meta-analysis.

Finally, 30 publications in PubMed (with free full text available online) were included in the final selection, including 12 review articles, 11 clinical trials, five randomized control trials, one observational study and one cohort study.

The maximum number of subjects in a study was 232, and the minimum was 14, and the total number of subjects included in all 30 studies was 951. All the documents reviewed were readily accessible for analysis, and the citations for the borrowed definitions were valid. A qualitative analysis was conducted on the existing data after inclusion/exclusion to provide the relevant disease and population with the necessary result.

Discussion

In the presented literature review, we found that FMT is helpful in treating patients with rCDIs. Moreover, studies have proved that FMT has provided an initial cure rate of nearly 90% in patients with rCDI [17,18]. Nonetheless, the optimal treatment option is still depending on the severity of the disease.

The normal intestinal microflora is a significant protective barrier against CDI. Bile acids play an essential part in the induction of *C. difficile* spore colonization in the intestine. Generally, primary bile acids stimulate* C. difficile* spores colonization, and the secondary bile acids inhibit this process. Typically, a greater level of secondary bile acids is present in the feces of healthy individuals compared to CDI. In contrast, primary bile acid level was more elevated in patients with recurrent CDIs compared to patients with their first episode of CDI. FMT induces the restoration of the intestinal microbiota that metabolizes the primary bile acids and the normalization of the secondary bile acids. Various cytokines such as IL-8, IL-1β, IL-6, TNFα, INFγ, and leukotriene B4 play a role in the pathogenesis of CDI. Moreover, *C. difficile* produces toxin A, “enterotoxin A” and toxin B, “cytotoxin B.” In severe situations, microulcerations are seen on the intestinal mucosa surface, covered with pseudomembranes [19,20].

Symptoms of rCDIs appear mostly during the first week after completion of the first episode of CDI treatment [21]. The clinical features of CDI range from the asymptomatic carrier state to life-threatening fulminant colitis. In addition to watery diarrhea, other features include fever, nausea and vomiting, abdominal pain, weakness, and anorexia. Although there is no active bleeding, stool for occult blood is usually positive. The symptoms vary according to the severity of the disease. Other serious complications include toxic megacolon, intestinal perforation and ileus, renal failure, systemic inflammatory response syndrome, septicemia, and death [22].

**Figure 1:**
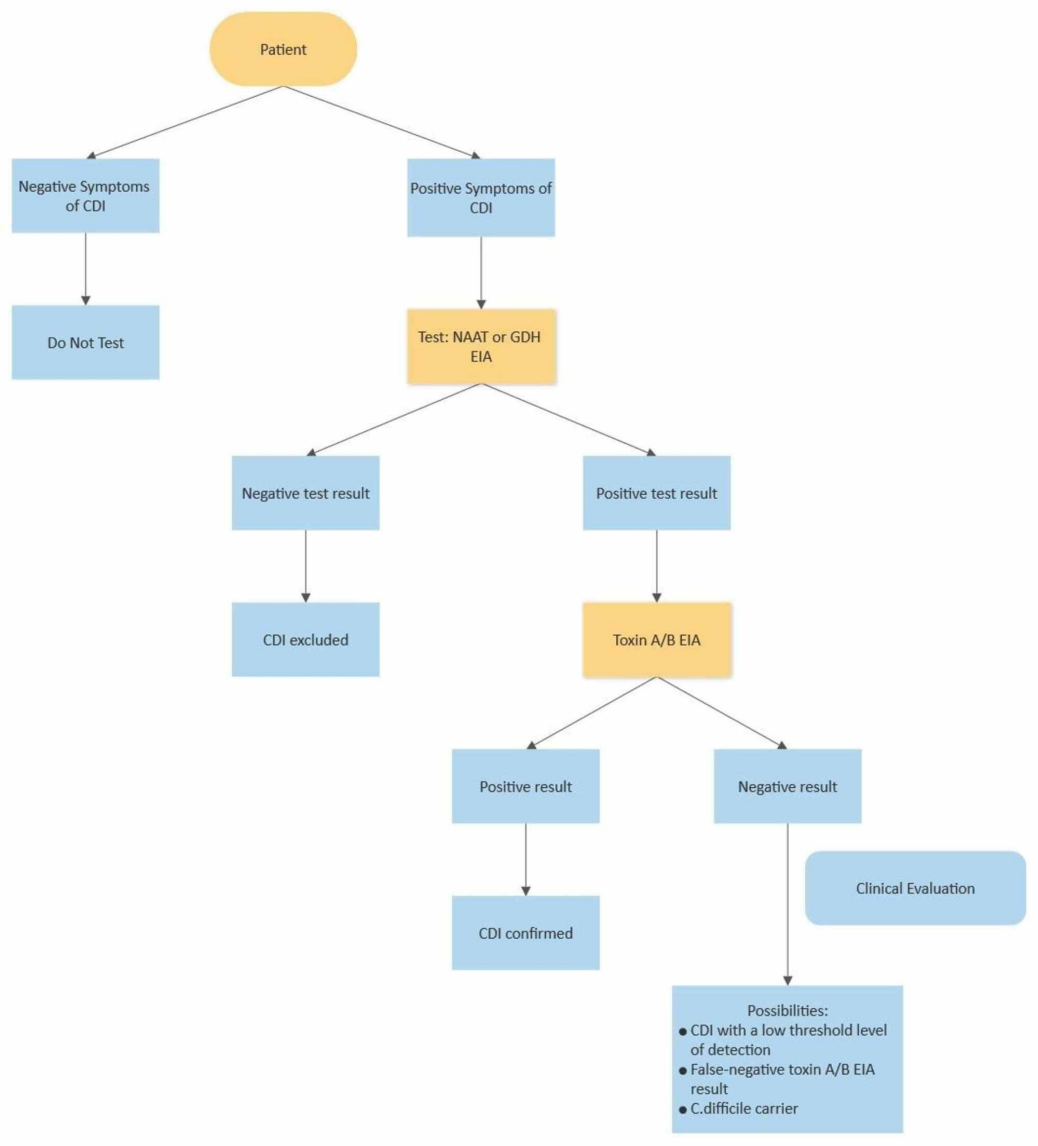
Flow chart of CDI diagnosis CDI: *Clostridium difficile* infection; *C. difficile*: *Clostridium difficile*; EIA: enzyme immunoassay; GDH: glutamate dehydrogenase; NAAT: nucleic acid amplification test

Figure 1 below represents the flow chart of CDI diagnosis [19].

Following the initial diagnosis and the treatment of CDI, the diagnosis of recurrent CDI is challenging. It requires the gathering of a detailed and complete clinical history of antibiotic use and symptom response. Patients should express a clinical response to antibiotics against *C. difficile*, then encounter a recurrence of prior symptoms within eight weeks of antibiotics cessation [23].

Table 3 exhibits the treatment options for recurrent *Clostridium difficile* infection [6].

**Table 3:** Treatment of recurrent Clostridium difficile infection Source: Adapted from Surawicz et al. [6]

Episode	Therapy
First recurrence	(1) Mild to moderate CDI: metronidazole 500 mg orally three times a day for 10 days, vancomycin 125 mg orally four times a day for 10 days, fidaxomicin 200 mg orally two times a day for 10 days (2) Severe CDI: vancomycin 125 mg orally four times a day for 10 days, fidaxomicin 200 mg orally two times a day for 10 days
Second recurrence	Tapered and/or pulsed vancomycin regimen fidaxomicin 200 mg orally two times a day for 10 days
Third or more recurrence	Fecal microbiota transplant (FMT) fidaxomicin 200 mg orally two times a day for 10 days

The three main indications for treatment with FMT are:

1. Recurrent *C. difficile* infection where there were (a) three or more episodes of mild to moderate *C. difficile* infection and failure of a six- to eight-week cycle with vancomycin, with or without an alternative antibiotic (rifaximin, nitazoxanide, or fidaxomicin), or (b) at least two episodes of *C. difficile* infection resulting in hospitalization

2. Moderate *C. difficile* infection not responding to standard therapy (vancomycin or fidaxomicin) for at least one week

3. Severe *C. difficile* infection (including fulminant) with no response to standard treatment after two days [24].

The routes of FMT administration are heterogeneous and include the upper GI tract (via esophagogastroduodenoscopy, nasogastric, or nasojejunal catheter or by ingestion of oral capsules) and the lower GI tract (by colonoscopy in the proximal colon, by enema and rectosigmoidoscopy in the distal colon, or a combined approach). The nasogastric route is efficient and safe for patients with contraindications to the colonoscopic route, which is well acknowledged. The massive burden is the vomiting and aspiration of the infused fecal contents. The enema route granted a significant degree of symptoms resolution. Nonetheless, it was mandatory to repeat the procedure in most cases until securing clinical improvement. Although the efficacy of retention enema had been in a dilemma, Orenstein et al. have demonstrated that retention enema is more effective and safer than placebo in phase I and phase II recurrent CDI clinical trials [25]. FMT via colonoscopy has superiority in allowing direct visualization of the entire colon, infusion of a large volume of fecal material, and better retention than the enema. Still, it is relatively risky of perforation, expensive, and invasive procedures. Oral capsules can have the same resolution rate compared with other routes, but they need a longer time to clinical improvement. The pros of the oral capsules are cost-effective, secure storage, proven efficacy, few side effects, easy administration, patient comfort, noninvasiveness, and safety for critically ill patients [26]. Kao et al. demonstrated that FMT via oral capsules had comparable outcomes of administering by colonoscopy in recurrent CDI patients [27]. There have been an enormous number of studies to evaluate the different methods of FMT administration. Youngster et al. conducted a randomized pilot study comparing upper and lower GI routes of FMT, and found a non-significant difference in clinical resolution rate between those two routes [28]. In a nutshell, currently, there is no firm evidence of the most favorable FMT administration method in the clinical setting, and the appropriate way should rely on the condition of the individual.

Even though the adverse reactions to FMT are rare, there are three adverse events of fecal microbiota transplantation.

1. Minor: Gastrointestinal adverse reactions such as abdominal discomfort, nausea/vomiting (especially with oral FMT route), diarrhea/constipation, bloating, flatulence, and transient fever

2. Severe: bleeding and perforation due to complications of endoscopy, aspiration due to sedation, pneumonia, infection, and sepsis

3. Additionally, the risk of potential transmission of blood-borne pathogens (e.g., hepatitis B & C, HIV), induction of chronic diseases due to alterations in the gut microbiota (e.g., obesity, diabetes, atherosclerosis, IBD, IBS, and colon cancer) [25].

Table 4 provides some details of randomized control trials for FMT administration and outcomes.

**Table 4:** Details of randomized control trials for FMT administration and outcomes

Author/ Publication year	Country	Study Design	Population	Sample Size	Main points
Lee et al., 2016 [29]	Canada	Randomized clinical trial	A total of 219 Canadian with rCDI at six academic medical centers	219	The study found out that subjects with recurrent or refractory CDI, the use of frozen compared with fresh FMT did not result in a worse proportion of clinical resolution of diarrhea over 13 weeks.
Kao et al., 2017 [27]	Canada	Randomized clinical trial	A total of 116 adult patients with RCDI were enrolled	116	The study discovered that among adults with rCDI, FMT via oral capsules was not inferior to delivery by colonoscopy for preventing recurrent infection over 12 weeks.
Kelly et al., 2016 [30]	United States	Randomized controlled trial	The study population comprised 46 adult outpatients who had ≥3 CDI recurrences and received a full course of vancomycin for their most recent acute episode	46	FMT using fresh donor stool delivered by colonoscopy after a course of vancomycin was effective at preventing further CDI episodes in patients with multiply recurrent infection.
Cammarota et al., 2015 [31]	Italy	Randomized controlled clinical trial	Patients who had recurrence CDI after ≥1 course of specific antibiotic therapy (at least 10 days of vancomycin 125 mg four times daily or at least 10 days of metronidazole 500 mg three times daily)	39	FMT by colonoscopy achieved significantly higher remission rates of recurrent CDI compared to vancomycin treatment.
Bruno et al., 2018 [24]	Brazil	Review	N/A	N/A	One of the main factors for frequent recurrences is due to the depletion of butyrate-producing bacteria which against C. difficile colonization in the intestinal mucosa. The 2013 C. difficile treatment guidelines of the American College of Gastroenterology suggest FMT as an alternative treatment for recurrent CDI that were resistant to a vancomycin treatment regimen.

## Conclusions

The current literature review concluded that the treatment of recurrent CDI is still challenging even though many treatment options are being researched and tested. Nevertheless, FMT is one of the most effective approaches and is becoming a more commonly used therapeutic option for managing multiple rCDIs. Currently, there is no firm evidence of the most favorable FMT administration method in the clinical setting, and the appropriate practice should be focused solely on the individual's condition. Therefore, more research is required in this area before it can be recommended on a routine basis.

## References

[1] Oren A, Garrity GM (2017). List of new names and new combinations previously effectively, but not validly, published. Int J Syst Evol Microbiol.

[2] Lessa FC, Mu Y, Bamberg WM (2015). Burden of Clostridium difficile infection in the United States. N Engl J Med.

[3] Dubberke ER, Olsen MA (2012). Burden of Clostridium difficile on the healthcare system. Clin Infect Dis.

[4] Cohen S, Gerding D, Johnson S, Kelly C (2010). Clinical practice guidelines for Clostridium difficile infection in adults. Infect Control Hosp Epidemiol.

[5] Debast SB, Bauer MP, Kuijper EJ (2014). European society of clinical microbiology and infectious diseases: update of the treatment guidance document for Clostridium difficile infection. Clin Microbiol Infect.

[6] Surawicz CM, Brandt LJ, Binion DG (2013). Guidelines for diagnosis, treatment, and prevention of Clostridium difficile infections. Am J Gastroenterol.

[7] Tang-Feldman Y, Mayo S, Silva J, Jr Jr, Cohen SH (2003). Molecular analysis of Clostridium difficile strains isolated from 18 cases of recurrent Clostridium difficile-associated diarrhea. J Clin Microbiol.

[8] McFarland LV, Surawicz CM, Rubin M, Fekety R, Elmer GW, Greenberg RN (1999). Recurrent Clostridium difficile disease: epidemiology and clinical characteristics. Infect Control Hosp Epidemiol.

[9] Doh YS, Kim YS, Jung HJ (2014). Long-term clinical outcome of Clostridium difficile infection in hospitalized patients: a single center study. Intest Res.

[10] Leffler DA, Lamont JT (2015). Clostridium difficile infection. N Engl J Med.

[11] Dubberke ER, Olsen MA (2012). Burden of Clostridium difficile on the healthcare system. Clin Infect Dis.

[12] Wilson K (1993). The microecology of Clostridium difficile. Clin Infect Dis.

[13] Broecker F, Kube M, Klumpp J (2013). Analysis of the intestinal microbiome of a recovered Clostridium difficile patient after fecal transplantation. Digestion.

[14] Seekatz A, Aas J, Gessert C, Rubin T, Saman D, Bakken J, Young V (2014). Recovery of the gut microbiome following fecal microbiota transplantation. mBio.

[15] Weingarden A, Chen C, Bobr A, Yao D, Lu Y, Nelson V (2014). Microbiota transplantation restores normal fecal bile acid composition in recurrent Clostridium difficile infection. Am J Physiol Gastrointest Liver Physiol.

[16] Cammarota G, Ianiro G, Gasbarrini A (2014). Fecal microbiota transplantation for the treatment of Clostridium difficile infection: a systematic review. J Clin Gastroenterol.

[17] Brandt LJ, Aroniadis OC, Mellow M (2012). Long-term follow-up of colonoscopic fecal microbiota transplant for recurrent Clostridium difficile infection. Am J Gastroenterol.

[18] Mattila E, Uusitalo-Seppälä R, Wuorela M (2012). Fecal transplantation, through colonoscopy, is effective therapy for recurrent Clostridium difficile. Gastroenterology.

[19] Jacek Czepiel, Mirosław Dróżdż, Hanna Pituch (2019). Clostridium difficile infection: review. Eur J Clin Microbiol Infect Dis.

[20] McDonald LC, Gerding DN, Johnson S (2018). Clinical practice guidelines for Clostridium difficile infection in adults and children: 2017 update by the Infectious Diseases Society of America (IDSA) and Society for Healthcare Epidemiology of America (SHEA). Clin Infect Dis.

[21] Moore SC (2018). Clostridium difficile: more challenging than ever. Crit Care Nurs Clin North Am.

[22] Bagdasarian N, Rao K, Malani PN (2015). Diagnosis and treatment of Clostridium difficile in adults: a systematic review. JAMA.

[23] Liubakka A, Vaughn BP (2016). Clostridium difficile infection and fecal microbiota transplant. AACN Adv Crit Care.

[24] Messias BA, Franchi BF, Pontes PH, Barbosa DÁ, Viana CA (2018). Fecal microbiota transplantation in the treatment of Clostridium difficile infection: state of the art and literature review. Rev Col Bras Cir.

[25] Orenstein R, Dubberke E, Hardi R (2016). Safety and durability of RBX2660 (microbiota suspension) for recurrent Clostridium difficile infection: results of the PUNCH CD study. Clin Infect Dis.

[26] Wang JW, Kuo C, Kuo F (2019). Fecal microbiota transplantation: review and update. J Formos Med Assoc.

[27] Kao D, Roach B, Silva M (2017). Effect of oral capsule-vs colonoscopy-delivered fecal microbiota transplantation on recurrent Clostridium difficile infection: a randomized clinical trial. JAMA.

[28] Youngster I, Sauk J, Pindar C (2014). Fecal microbiota transplant for relapsing Clostridium difficile infection using a frozen inoculum from unrelated donors: a randomized, open-label, controlled pilot study. Clin Infect Dis.

[29] Lee CH, Steiner T, Petrof EO (2016). Frozen vs fresh fecal microbiota transplantation and clinical resolution of diarrhea in patients with recurrent Clostridium difficile infection: a randomized clinical trial. JAMA.

[30] Kelly CR, Khoruts A, Staley C (2016). Effect of fecal microbiota transplantation on recurrence in multiply recurrent Clostridium difficile infection: a randomized trial. Ann Intern Med.

[31] Cammarota G, Masucci L, Ianiro G (2015). Randomised clinical trial: fecal microbiota transplantation by colonoscopy vs. vancomycin for the treatment of recurrent Clostridium difficile infection. Aliment Pharmacol Ther.

